# Population genomics of the basket cockle *Clinocardium nuttallii* in the southern Salish Sea: Assessing genetic risks of stock enhancement for a culturally important marine bivalve

**DOI:** 10.1111/eva.13359

**Published:** 2022-03-08

**Authors:** James L. Dimond, Ryan N. Crim, Elizabeth Unsell, Viviane Barry, Jodie E. Toft

**Affiliations:** ^1^ 1632 Puget Sound Restoration Fund Bainbridge Island Washington USA; ^2^ 1632 Shannon Point Marine Center Western Washington University Anacortes Washington USA; ^3^ 265912 Fisheries Department Suquamish Tribe Suquamish Washington USA

**Keywords:** *Clinocardium*, population connectivity, RADseq, Salish Sea, stock enhancement, subsistence

## Abstract

Coastal Indigenous communities that rely on subsistence harvests are uniquely vulnerable to declines in nearshore species. The basket cockle *Clinocardium nuttallii* is among the favored foods of Indigenous people along the northwest Pacific coast of North America, yet localized declines in their abundance have led to interest in stock enhancement efforts. We used a population genomics approach to examine potential risks associated with stock enhancement of *C. nuttallii* in the southern Salish Sea, a large inland estuary that includes Puget Sound. More than 8000 single nucleotide polymorphisms across 349 individuals at 12 locations were assembled de novo using restriction site‐associated DNA sequencing. Results indicated that *C. nuttallii* within the southern Salish Sea were distinct from those along the outer Pacific coast (*F*_ST_ = 0.021–0.025). Within the southern Salish Sea, *C. nuttallii* populations appear to be well‐connected despite numerous potential impediments to gene flow; Hood Canal, which experiences the lowest flushing rates of all Puget Sound sub‐basins, was a minor exception to this strong connectivity. We found evidence of isolation by distance within the southern Salish Sea, but the slope of this relationship was shallow, and *F*_ST_ values were low (*F*_ST_ = 0.001–0.004). Meanwhile, outlier analyses did not support the hypothesis that southern Salish Sea sub‐populations are locally adapted. Estimates of effective population size had no upper bound, suggesting potentially very high adaptive capacity in *C. nuttallii*, but also making it difficult to assess potential reductions in effective population size resulting from stock enhancement. We present several strategies to augment cockle populations for subsistence harvest that would limit risk to the genetic diversity of wild cockle populations.

## 1 INTRODUCTION

Shellfish have been an important food for people throughout the world for eons, as evidenced by the unearthing of shell middens on nearly every continent (Álvarez et al., 2011). In the Salish Sea, a large inland estuary along the northern Pacific coast of North America, Coast Salish people historically built and tended intertidal clam gardens, which are regarded as an ancient form of aquaculture and provide further testament to the nutritional and cultural importance of shellfish for Indigenous people (Groesbeck et al., 2014; Poe et al., 2016). Today, Coast Salish people continue to forage for shellfish as a primary means of subsistence (Poe et al., 2016). Unfortunately, however, several native shellfish species in the region have been severely depleted, such as Olympia oyster (White et al., 2009), pinto abalone (Carson & Ulrich, 2019), and native littleneck clam (Barber et al., 2019), threatening the persistence of the species at risk as well as the livelihoods of Indigenous people.

Among the principal shellfish species harvested by Coast Salish people is the basket or heart cockle, *Clinocardium nuttallii* (Conrad, 1837). This species ranges widely along the Pacific coast of North America, from southern California to western Alaska, and has also been found on the island of Hokkaido (Kilmer, 1973). It is a broadcast spawning simultaneous hermaphrodite, producing planktotrophic larvae with a duration up to approximately 10 days (Gallucci & Gallucci, 1982; Liu et al., 2010). Cockles populate low intertidal and shallow subtidal areas and have been reported at densities up to 232 g (~2–3 adult cockles) m^−3^ intertidally in the Salish Sea (Barber et al., 2019). In recent years, certain beaches have become closed to subsistence harvest due to population declines. Multi‐decadal surveys of intertidal clams in the southern Salish Sea have confirmed that cockles have declined on some beaches, while on other beaches their abundance has either increased or remained steady (Barber et al., 2019). Anecdotal evidence suggests that cockles may be susceptible to periodic localized die‐offs due to factors such as high temperatures and hypoxia, which are becoming increasingly common due to marine heatwaves and nearshore pollution (Khangaonkar et al., 2018; Oliver et al., 2018). Recently, widespread mortality of cockles was documented on beaches in the Salish Sea following an unprecedented summer heatwave that coincided with some of the lowest tides of the year (J. Barber, unpublished data). This has raised concerns among tribal fishers and fishery co‐managers about the ability of cockles on particular beaches to repopulate naturally, which could be related to factors such as low population connectivity or local adaptation.

Populations of marine species were once thought to be genetically homogenous, being highly interconnected by seemingly infinite populations of adults and larvae that could disperse over large expanses of water (Hauser & Carvalho, 2008). Over the last few decades, this notion has been overturned by modern genetic data indicating that a surprising number of marine species exhibit significant population structure, sometimes at relatively small spatial scales (Hauser & Carvalho, 2008). Causes of population structure in marine species include processes such as local retention of larvae, adaptation to local environments, spatial and temporal reproductive isolation, and high variance in reproductive success (Hauser & Carvalho, 2008). In the southern Salish Sea, which includes Puget Sound and its various sub‐basins, many potential opportunities for restricted gene flow are created by its estuarine circulation, fjord‐like bathymetry, and tortuous geography.

Localized cockle population declines have spurred interest in using aquaculture to supplement beaches with hatchery‐spawned and raised juveniles. In fact, *C. nuttallii* has long been viewed as an attractive species for aquaculture development in the region, and initial research on captive spawning and larval rearing practices in the past decade have proven that *C. nuttallii* is highly amenable to aquaculture production (Dunham et al., 2013a, 2013b; Liu, Alabi, & Pearce, 2008a, 2008b; Liu et al., 2009, 2010, 2011). However, fishery co‐managers in Washington State have agreed that genetic risks associated with aquaculture must be addressed before it can be used as a restoration practice.

To evaluate potential genetic risks of aquaculture enhancement for *C. nuttallii*, we evaluated the population genomics of cockles from throughout the southern Salish Sea, as well as the outer Washington coast. We used restriction site‐associated DNA sequencing to provide a comprehensive picture of genomic variability, population connectivity, and potential local adaptation in this bivalve.

## 2 METHODS

**FIGURE 1:**
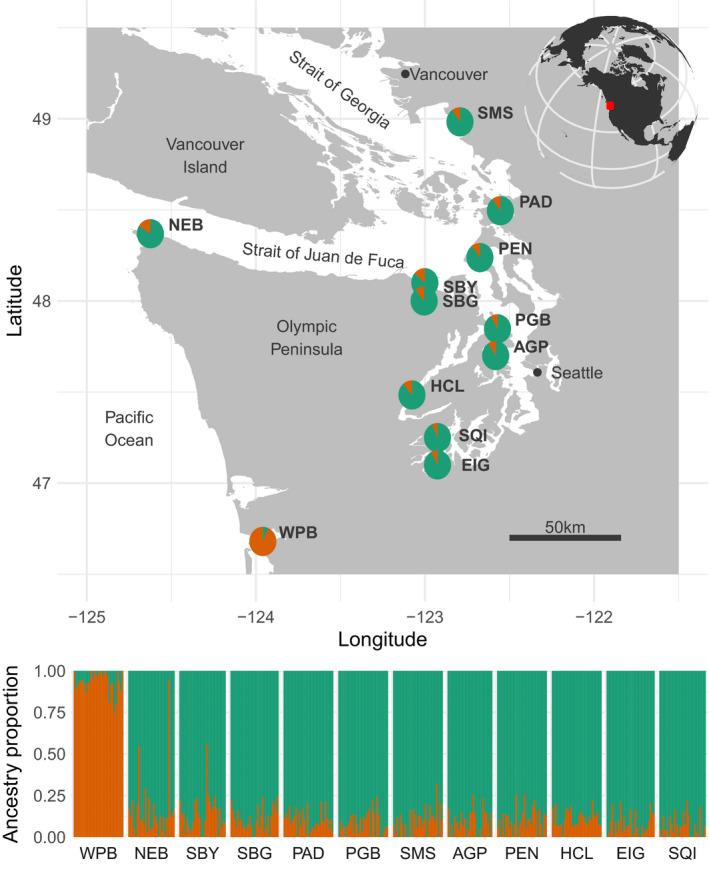
Upper panel: Map of the study area showing *Clinocardium nuttallii* collection sites in the southern Salish Sea and coastal Washington, with inset globe map in the upper right showing location of the study area highlighted in red. Individual pie charts show ancestry proportions at each site based on LEA analysis with *K* = 2 ancestral populations. Lower panel: Detailed view of ancestry components for each sample at each site. Sites are ordered by water distance from Willapa Bay

Cockles were collected intertidally (−0.15 to −0.55 m below mean lower low water) during low tides between October 2019 and January 2020. Locations of sampling sites were chosen to span a wide range of water bodies and sub‐basins in the southern Salish Sea and coastal Washington, including Puget Sound, the Strait of Juan de Fuca, and the Strait of Georgia (Figure 1). In addition to natural intertidal habitats, we collected cockles from inside geoduck (*Panopea generosa*) culture tubes at two sites. Cockles growing inside geoduck tubes are wild animals that have naturally recruited to these artificial habitats. One of the sites (Sequim Bay geoduck tubes) was within 50 m of a natural cockle collection habitat (Sequim Bay), while another (Eld Inlet geoduck tubes) was within 7 km of a natural collection habitat (Squaxin Island). At all sites, adult cockle specimens >~50 mm in length were targeted for collection, and specimens were kept in coolers with ice packs prior to dissection and tissue sampling within 12–48 h of collection.

### 2.1 Tissue collection, DNA extraction, library preparation, and sequencing

Tissue samples from cockles were taken by clipping small pieces of foot and mantle tissue and preserving them in 95% ethanol. Samples were kept at 4°C prior to DNA extraction, which took place within 1–3 months of collection. DNA was extracted using the Qiagen DNeasy Blood and Tissue 96‐well kit, following overnight lysis in Proteinase K. Mantle tissue was chosen over foot tissue for all samples based on slightly higher DNA quality in initial extractions as assessed by agarose gel electrophoresis. Samples were shipped to Eurofins Diagnostics (River Falls, WI, USA) for DNA quantitation and normalization, and then to Floragenex Inc. (Portland, OR, USA) for RADseq library preparation (Baird et al., 2008) with the restriction enzyme *SbfI*. A total of 380 samples, including 20 technical replicates sequenced in duplicate, were allocated randomly across four libraries, which were each sequenced on three lanes of an Illumina HiSeq producing paired‐end, 100 bp reads.

### 2.2 Bioinformatic processing and filtering

Sequencing reads were demultiplexed with the Stacks v2.55 *process_radtags* program (Rochette et al., 2019). Demultiplexed reads were then assembled de novo with the Stacks v2.55 pipeline using the *denovo_map.pl* program. Prior to running the pipeline on the entire set of samples, a range of assembly parameters were tested on a subset of samples using the *r80* method (Paris et al., 2017; Rochette & Catchen, 2017). Based on these results, we chose *M* = 4 and *n* = 4 for the full analysis. The full analysis program settings included the PCR duplicate removal option, a minimum minor allele count of 3 to process a locus, and the requirement that a locus occurred in a minimum of 70% of individuals in a population in order to process it. Subsequent SNP filtering of the resulting Stacks VCF file was done with the R package *vcfR* v1.10.0 (Knaus & Grünwald, 2017). Variants with sequencing depth outside of 95% confidence intervals were excluded, as were those with quality scores below 20 and with greater than 20% missing data. To exclude variants according to linkage disequilibrium (LD), LD‐based SNP pruning was performed with the R package *SNPRelate* v1.20.1 (Zheng et al., 2012) using an LD threshold of *r* ^2^ = 0.2. Lastly, we tested for the presence of paralogous loci in the assembly using the proportion of heterozygotes and deviations in read ratios procedure described by McKinney et al. (2017); we reasoned that this would assist in identifying potential over‐clustering of loci in the assembly. We did not apply Hardy–Weinberg equilibrium (HWE) filtering for two reasons. First, recent work by Pearman et al. (2021) examining HWE filtering found limited support for its efficacy. Second, HWE is most commonly employed to reduce genotyping errors. Given that genotyping error as assessed by technical replicates was extremely low, we did not feel that it was necessary to implement additional filters.

Genotyping error was assessed in the final SNP dataset based on SNP mismatches between 18 samples sequenced in duplicate serving as technical replicates; 2 of the initial 20 replicates were dropped during filtering due to low coverage. Technical replicates were removed from the dataset for subsequent analyses.

### 2.3 SNP outlier tests

To identify outlier SNP markers, we used two different programs. The R package *pcadapt* v4.3.3 (Luu et al., 2017) was used to detect outliers based on principal components analysis. The R package *OutFLANK* v0.2 (Whitlock & Lotterhos, 2015) was then used to detect outliers based on the *F*_ST_ distribution. Loci in consensus across these two methods were separated as outlier loci for subsequent analyses.

### 2.4 SNP analysis

Population statistics, including gene diversity, observed heterozygosity, inbreeding coefficients, and genetic differentiation, were computed with the R package *hierfstat* v0.04–22 (Goudet, 2005). Pairwise *F*_ST_ was computed according to the method of Weir and Cockerham (1984) as implemented in *hierfstat* and *StAMPP* v1.6.1 (Pembleton et al., 2013), with 95% confidence intervals and *p* values computed with *StAMPP*. Population differentiation was first examined without a priori population groups using the R package *LEA* v3.6.0 (Frichot & François, 2015); admixture was tested with a range of *K* = 1–10, and cross‐entropy values were used to evaluate optimal *K*. Collection sites were then used as a priori groups for discriminant analysis of principal components (DAPC) with the R package *adegenet* v2.1.3 (Jombart, 2008). To determine the optimal number of principal components to include in the DAPC, cross‐validation was implemented with the *xvalDapc* function in *adegenet* using default parameters of 0.9 for the training set and 30 replicates.

Isolation by distance was tested by creating a pairwise matrix of lineal water distances between sites. The distance matrix and the *F*_ST_ matrix were compared with a Mantel test using the R package *ape* v5.3 (Paradis & Schliep, 2019); to compare Willapa Bay and Salish Sea sites, for which full distance matrices were not relevant, Pearson correlation was used.

Relatedness between individuals was assessed using the genomic relatedness estimator of Yang et al. (2010) implemented in *StAMPP* (Pembleton et al., 2013). The effective population size was computed using the linkage disequilibrium method with the program *NeEstimator* v2.1 (Do et al., 2014).

## 3 RESULTS

### 3.1 Cockle size and approximate age classes

Cockles collected at each site generally spanned a range of shell lengths, corresponding to multiple age classes based on age‐size data from San Juan Island, Washington, reported by Gallucci and Gallucci (1982). These size ranges suggest that the majority of cockles collected were in the 2‐ to 4‐year age range (Figure S1). One notable exception was cockles collected from Eld Inlet geoduck tubes, which were composed almost entirely of a putative 2‐year‐old age class.

### 3.2 SNP data

After all filtering procedures, the final SNP dataset consisted of 8674 variants and 367 samples, with 13% missing data. No paralogous loci were identified based on the read ratio deviation versus proportion of heterozygotes procedure (Figure S2). Based on technical replicates sequenced in duplicate, mean genotyping error of the final assembly was 0.72% (Figure S3); in other words, 99.28% of SNPs were called identically between two identical samples. Final sample sizes included in the analysis ranged from 27 to 30 individuals per site (Table 1). All sites exhibited similar levels of observed (*H*_o_; 0.0722–0.0804) and expected heterozygosity (*H*_e_; 0.0695–0.0763), and no sites showed heterozygote deficiencies. The highest and lowest observed and expected heterozygosities were in Willapa Bay and Port Gamble Bay, respectively. Allelic richness was similar among populations; however, the number of private alleles was much higher among Willapa Bay cockles, followed by Hood Canal (Table 1).

**TABLE 1:** Site names and codes, sample sizes, observed heterozygosity (*H*_o_), expected heterozygosity (*H*_e_), inbreeding coefficients (*F*_IS_), allelic richness, and private alleles for the *Clinocardium nuttallii* samples included in the final assembly

Site	N	*H*_o_	*H*_e_	*F*_IS_	Allelic richness	Private alleles
Hood Canal (HCL)	30	0.0748	0.0704	−0.0238	1.400	36
Agate Pass (AGP)	27	0.0752	0.0724	−0.0108	1.414	17
Neah Bay (NEB)	28	0.0725	0.0703	−0.0081	1.401	17
Penn Cove (PEN)	30	0.0745	0.0710	−0.0185	1.407	14
Port Gamble Bay (PGB)	30	0.0722	0.0695	−0.0118	1.402	22
Sequim Bay (SBY)	28	0.0749	0.0709	−0.0221	1.405	18
Sequim Bay geoduck (SBG)	29	0.0733	0.0697	−0.0173	1.401	10
Padilla Bay (PAD)	30	0.0726	0.0698	−0.0115	1.401	14
Semiahmoo Spit (SMS)	30	0.0740	0.0716	−0.0105	1.412	25
Eld Inlet geoduck (EIG)	29	0.0735	0.0702	−0.0156	1.404	20
Squaxin Island (SQI)	28	0.0726	0.0699	−0.0130	1.403	9
Willapa Bay (WPB)	30	0.0804	0.0763	−0.0193	1.421	172

### 3.3 Population structure

**FIGURE 2:**
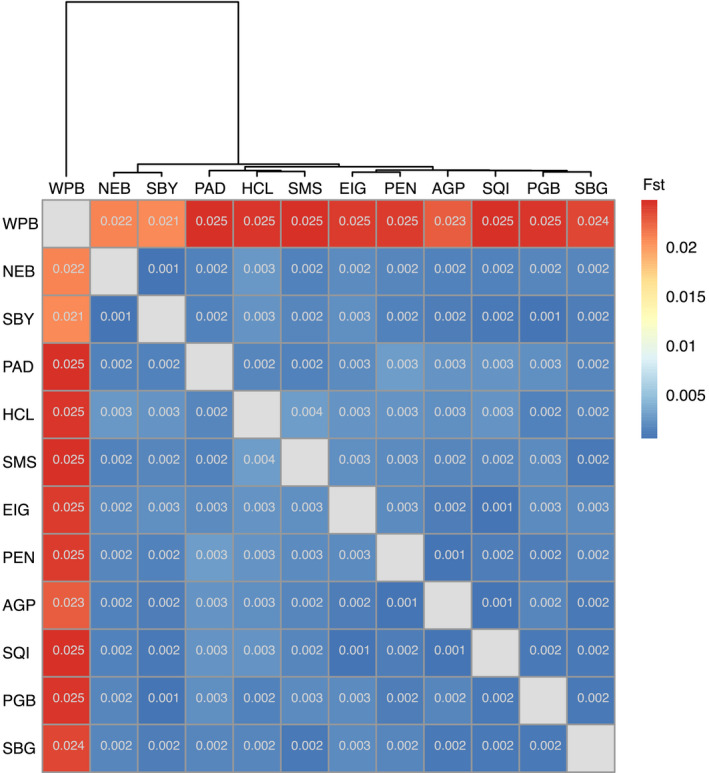
Pairwise *F*_ST_ matrix with populations ordered by hierarchical clustering of *F*_ST_ values. All *F*_ST_ values were significantly different from zero

Admixture analysis showed that *K* = 2 had only slightly higher cross‐entropy than *K* = 1, with subsequent values of *K* showing sharp increases in cross‐entropy (Figure S4). We therefore plotted admixture components at *K* = 2, which indicated a strong population break between Salish Sea sites and Willapa Bay, on the outer Pacific coast (Figure 1). In corroboration, pairwise *F*_ST_ values indicated that divergence of Willapa Bay cockles from all other sites was an order of magnitude higher (*F*_ST_ = 0.022–0.025) than divergence of remaining sites from each other (*F*_ST_ = 0.001–0.004; Figure 2). Of these remaining sites, hierarchical clustering showed that Neah Bay and Sequim Bay were the least divergent from Willapa Bay (top of Figure 2). After Willapa Bay, Hood Canal had the second highest *F*_ST_ values, suggesting that Hood Canal exhibited a small degree of differentiation from other Salish Sea sites. All pairwise *F*_ST_ values were significantly different from zero at α = 0.05.

**FIGURE 3:**
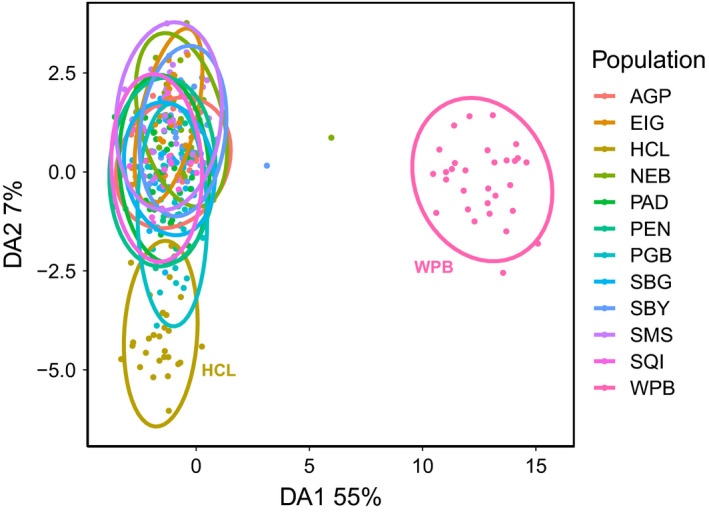
Discriminant analysis of principal components (DAPC) scatterplot. The percentage of DAPC variance captured by each axis is shown in axis labels

These results were mirrored by DAPC analysis. The *xvalDapc* function in *adegenet* determined that 150 principal components were associated with the lowest cross‐validation error (Figure S5). Sixty percent of the variance was explained by a DAPC with 150 principal components. The majority of the DAPC variance (55%) was explained by the first discriminant function (Figure 3). Along this first discriminant axis, Willapa Bay exhibited clear divergence from Salish Sea sites (Figure 3). Along the second discriminant axis (7% of variance), Hood Canal showed a less pronounced degree of differentiation from the remaining sites.

### 3.4 Neutral vs. outlier loci

**FIGURE 4:**
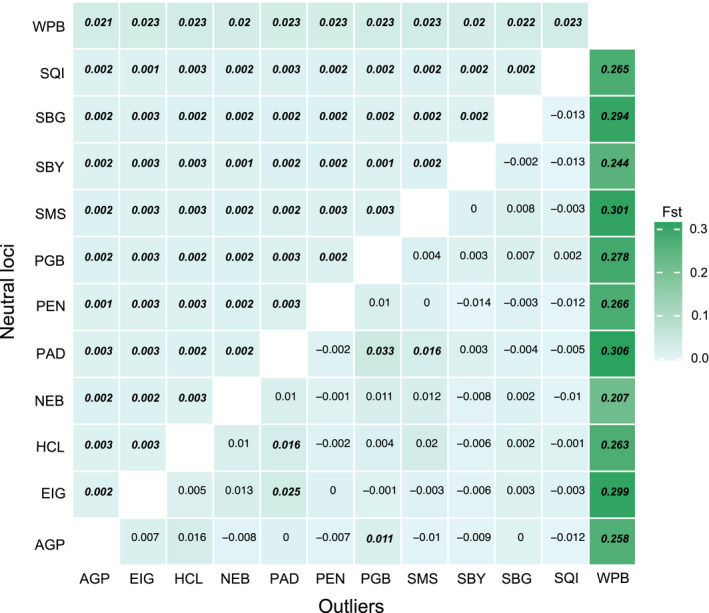
Pairwise *F*_ST_ based on 8661 neutral (upper triangle) and 13 outlier (lower triangle) loci. Significant *F*_ST_ values are shown in bold italics

*Pcadapt* and *OutFLANK* identified 38 and 14 SNP outliers, respectively; 13 of these were in consensus across both methods. Pairwise *F*_ST_ based only on outliers indicated much more pronounced differentiation between Willapa Bay and all Salish Sea sites compared to the 8661 neutral markers (Figure 4). However, *F*_ST_ values between Willapa Bay and all Salish Sea sites based on neutral markers were still an order of magnitude higher than pairwise differentiation within Salish Sea sites.

### 3.5 Isolation by distance

**FIGURE 5:**
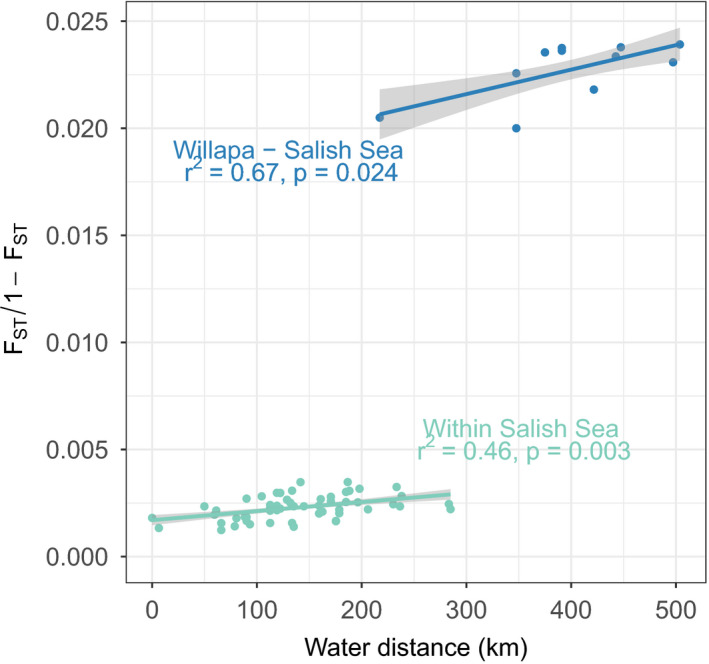
Tests of isolation by distance in *Clinocardium nuttallii*. Pairwise genetic differentiation and water distances within the Salish Sea were tested separately from those between Willapa Bay and the Salish Sea. Results of a Mantel test are shown for the within Salish Sea analysis, while results of a Pearson correlation are shown for Willapa Bay vs. Salish Sea

Analysis of isolation by distance using the neutral SNP dataset revealed an overall positive relationship between pairwise water distance and *F*_ST_ (Figure 5). However, the relationship was highly disjunct due to the order of magnitude higher *F*_ST_ between Willapa Bay and Salish Sea sites compared to sites within the Salish Sea. For this reason, the analysis was separated between Willapa Bay vs. Salish Sea comparisons and within Salish Sea comparisons. There was a significant increase in pairwise *F*_ST_ with increasing water distance both within the Salish Sea (Mantel test; *r* ^2^ = 0.46, *p* = 0.003) and between the Salish Sea and Willapa Bay (Pearson correlation; *r* ^2^ = 0.67, *p* = 0.024).

### 3.6 Relatedness

The genomic relatedness matrix indicated that no closely related individuals were found within a location or between distinct locations (Figure S6). Individuals at Willapa Bay exhibited the highest relatedness values, but these values were still too low to suggest inbreeding, which is also reflected by the low *F*_IS_ value for Willapa Bay (Table 1).

### 3.7 Effective population size

Estimates of *N_e_* based on the heterozygote excess method were in the range of 22.7–142.9, while for the linkage disequilibrium method estimates were infinite (Table 2).

**TABLE 2:** Effective population size (*N_e_*) based on the heterozygote excess method (*N_e_Ht*) and linkage disequilibrium (*N_e_LD*) method, for minimum allele frequencies of 0.01

Population	N_e_Ht (95% CI)	N_e_LD (95% CI)
Hood Canal	67.6 (44.4–142.9)	∞ (∞–∞)
Agate Pass	22.7 (19.5–27.4)	∞ (∞–∞)
Neah Bay	28.9 (23.9–36.6)	∞ (∞–∞)
Penn Cove	24.3 (20.9–29.2)	∞ (∞–∞)
Port Gamble Bay	31.3 (25.5–40.7)	∞ (∞–∞)
Sequim Bay	51.4 (37–84.4)	∞ (∞–∞)
Sequim Bay geoduck	34.1 (27.2–45.8)	∞ (∞–∞)
Padilla Bay	45.5 (33.8–69.9)	∞ (∞–∞)
Semiahmoo Spit	47 (34.3–75)	∞ (∞–∞)
Eld Inlet geoduck	49.9 (35.9–82)	∞ (∞–∞)
Squaxin Island	41.6 (31.7–60.9)	∞ (∞–∞)
Willapa Bay	28.4 (23.4–36)	∞ (∞–∞)

## 4 DISCUSSION

Our results indicate that *C. nuttallii* within the southern Salish Sea are distinct from those in Willapa Bay. Although we lack another outer coast population with which to corroborate this result, it is in agreement with the strong differentiation between coastal populations and those within the Salish Sea observed among other marine invertebrates and fishes (Buonaccorsi et al., 2005; Drinan et al., 2018; Iwamoto et al., 2015; Jackson & O’Malley, 2017; Silliman, 2019). Indeed, we found that cockles from Neah Bay, which is relatively close to Willapa Bay at the western limit of the Salish Sea (217 km), are much more similar to cockles from Squaxin Island, which is further away at the southern extent of Puget Sound (285 km). For Olympia oysters, Silliman (2019) concluded that the entrance to the Strait of Juan de Fuca is among the major barriers to gene flow along the Pacific coast of North America. This is likely due to oceanographic processes, principally the net outflow of Salish Sea surface waters through the Strait of Juan de Fuca resulting from estuarine circulation (Khangaonkar et al., 2017). Additionally, conditions within Willapa Bay may also restrict gene flow; modeling studies of green crab larval retention suggested that 5%–40% of larvae spawned during summer were retained within Willapa Bay (Banas et al., 2009). Although neutral processes appear to contribute most significantly to differentiation of Willapa Bay cockles, outlier loci were strongly associated with Willapa Bay, suggesting that adaptive processes may also play a role.

Within the southern Salish Sea, *C. nuttallii* appear to be a well‐connected metapopulation despite the numerous inlets, embayments, and other potential impediments to gene flow. Although we detected evidence of isolation by distance within the southern Salish Sea, the slope of this relationship was shallow, and *F*_ST_ values were low. Moreover, based on outlier analyses, our results do not support the hypothesis that sub‐populations are locally adapted. However, cockles within Hood Canal showed slight evidence of divergence from other sub‐populations. Similarly, differentiation of Hood Canal populations from those in other Puget Sound basins has been observed among Dungeness crab (Jackson & O’Malley, 2017) and yelloweye rockfish (Andrews et al., 2018). Among all Puget Sound sub‐basins, water in Hood Canal is retained for the longest duration (Ebbesmeyer, 1984), making larval retention perhaps the most likely explanation for divergence of Hood Canal populations. At the mouth of Hood Canal, deep water flow is limited by a relatively shallow sill at 50 m depth, while surface flow is partially inhibited by a floating bridge with a draft of 3.7 m spanning approximately 90% of the width of the inlet (Khangaonkar & Wang, 2013). This bridge has been shown to impede movement of steelhead smolt out of Hood Canal (Moore et al., 2013).

High connectivity and gene flow among Salish Sea cockle populations are further supported by relatively high observed heterozygosity, negative inbreeding coefficients, and low levels of relatedness among cockles in a given location. These attributes are particularly notable given that *C. nuttallii* is a simultaneous hermaphrodite capable of self‐fertilization. Liu et al. (2008b) found that some level of self‐fertilization appears to be unavoidable in *C. nuttallii*, and reported a rate of up to 95% self‐fertilization among newly released eggs in the laboratory; however, this rate dropped to 25.7% among 2–4 cell embryos, perhaps as a result of deleterious alleles. The reduced fitness of inbred individuals through the expression of deleterious alleles among homozygotes (inbreeding depression) is thought to strongly limit the influence of inbreeding (Charlesworth & Charlesworth, 1987), and it is possible that reductions in the survival and fitness of selfed *C. nuttallii* significantly diminish their proportion among surviving adults. For example, selfed larvae of the scallop *Argopecten circularis* exhibited 41.6% lower survival than larvae from pair‐mated crosses (Ibarra et al., 1995). Nonetheless, selfed individuals that survive and reproduce have the potential to significantly reduce the genetic diversity of the population. Perhaps this is reflected in the expected heterozygosity (gene diversity), which is notably low yet remarkably consistent across populations (0.0695–0.0763). However, relatively low gene diversity has also been observed in two other Atlantic cockle species, *Cerastoderma edule* and *C. glaucum* (Coscia et al., 2020; Sromek et al., 2019), both nonselfing, dioecious species. Although an excess of heterozygosity can be caused by over‐clustering of loci during de novo catalog construction (O’Leary et al., 2018), no paralogs were detected by the proportion of heterozygotes and deviations in read ratios procedure (McKinney et al., 2017), and the extremely low genotyping error provides additional evidence against over‐clustering.

By contrast with the heterozygote excess and negative *F*_IS_ we found in *C. nuttallii*, heterozygote deficiencies are common among bivalves and were observed in recent SNP‐based analyses of both of the Atlantic cockle species mentioned above, as well as in Olympia and eastern oysters (Bernatchez et al., 2019; Coscia et al., 2020; Silliman, 2019; Sromek et al., 2019). The heterozygote excess observed here among *C. nuttallii* is particularly unusual in light of the potential for selfing. It suggests that *C. nuttallii* has evolved mechanisms to overcome the effects of selfing, such as through strong selection against recessive alleles expressed in homozygotes (heterozygote advantage) and resulting associative overdominance (Ohta, 1971; Ohta & Cockerham, 1974). Additionally, the ~10‐day larval period is likely to allow dispersal of any potential selfed offspring far from parents, and indeed, the high connectivity of *C. nuttallii* observed here is consistent with strong dispersal.

The high population connectivity, and by inference, effective dispersal among southern Salish Sea *C. nuttallii* populations suggests that natural repopulation of beaches following localized disturbances should occur quickly. Moreover, since localized die‐offs are thought to primarily affect intertidal populations, subtidal *C. nuttallii* populations represent refuges that may facilitate reseeding of adjacent intertidal areas even in the case of limited larval supply from other locales (Ratti, 1977). In an Oregon estuary, for example, Ratti (1977) found that although subtidal populations were lower than those in the intertidal zone, subtidal individuals were more fecund and grew faster. However, repopulation of intertidal areas does not always occur quickly based on both anecdotal and quantitative evidence (Barber et al., 2019). Notably, while two other intertidal clam species in the region, *Leukoma staminea* and *Saxidomus gigantea*, showed multi‐year population synchrony throughout the southern Salish Sea, *C. nuttallii* population trends were beach specific (Barber et al., 2019). Together with our evidence that connectivity of *C. nuttallii* is strong, this suggests that factors other than larval supply, such as postsettlement survival or changes in habitat quality or suitability, may be responsible for population declines in certain areas. For example, large‐scale die‐offs or heavy harvests of cockles could lead to alternative stable states (Beisner et al., 2003) that make it difficult for cockles to become reestablished. Alternatively, climate change may be slowly bringing conditions on some beaches above survival thresholds for cockles, which are not well buffered from air temperature extremes during low tides due to their relatively shallow sediment burial depths.

Supplementation of *C. nuttallii* populations using captive breeding may offer a solution to fluctuations or localized declines in abundance. The high connectivity and lack of evidence for locally adapted populations in the southern Salish Sea suggest potentially low genetic risks to wild stocks. On the other hand, effective population size (*N_e_*) estimates based on the LD method were infinite, which makes it difficult to estimate the genetic consequences of supplementation. Assessments of *N_e_* in large marine populations are notoriously problematic and may require extremely large sample sizes to obtain any degree of accuracy (Marandel et al., 2019). Although *N_e_* values based on the heterozygote excess method were finite and could be used to estimate the Ryman–Laikre effect, which reflects the potential increase in inbreeding and reduced effective population size as a result of supplementation (Ryman & Laikre, 1991; Waples et al., 2016), the potentially much larger *N_e_* as assessed by LD is more important, because populations with large *N_e_* are at greatest risk for increased inbreeding due to supplementation (Hare et al., 2011; Waples et al., 2016). Supportive breeding effectively increases variance in reproductive success, and for populations with large *N_e_*, maintaining large *N_e_* in the face of supplementation would require unreasonably large captive broodstocks (Hare et al., 2011). Furthermore, the lack of an upper bound on *N_e_* makes it impossible to calculate the potential change in *N_e_* resulting from a supplemental breeding program.

The most risk‐averse solution may be to ensure that captive‐bred individuals have limited reproductive contribution to wild stocks (Waples et al., 2016). This can be done by either supplementing wild populations with a very small number of captive‐bred animals (e.g., <10% of the wild population; Waples et al., 2016), by developing techniques to produce reproductively sterile animals, or by harvesting captive‐bred individuals before they become reproductively mature (Waples et al., 2016). For example, in support of the latter strategy, a lack of introgression between hatchery‐bred and wild Sydney rock oysters (*Saccostrea glomerata*) may have been attributed to harvest of hatchery‐bred individuals prior to reproductive maturity (Thompson et al., 2017). Supplemental hatchery‐bred *C. nuttallii* could be collected at an acceptable harvest size before they reproduce in spring or summer of their second year, when they reach a size of approximately 50 mm (Gallucci & Gallucci, 1982; Ratti, 1977; Table 3). This strategy employs standard shellfish aquaculture techniques and can be considered a form of sea farming; it is distinguished from stock enhancement due the removal of individuals before they can interbreed with wild stocks (Grant et al., 2017).

**TABLE 3:** Summary of the costs and benefits of strategies to augment populations of *Clinocardium nuttallii* for subsistence harvest

Augmentation strategy	Costs	Benefits	Notes
Translocation of cockles from geoduck tubes to beaches	Labor and transportation costs to collect and translocate cockles	No impact on wild *N_e_*. No hatchery production costs. Cockles in geoduck tubes would be removed anyway	Least risky approach from genomic standpoint
Containerized sea farming of hatchery‐produced cockles	Hatchery production costs. Cockles harvested at small size to avoid reproduction	Low projected impact on wild *N_e_*	Cockles collected at age 2, ~50 mm
Stock enhancement through beach seeding	Hatchery production costs. Potential for reduction in wild *N_e_*	Cockles harvested at larger size than with sea farming	Genetic risk increases with size of breeding program. Greater attention to broodstock sizes recommended

A potentially attractive sea farming‐based alternative to a hatchery breeding program would be to translocate wild cockles from geoduck culture tubes to beaches where they have declined. Cockles growing in geoduck culture tubes can be regarded as a nuisance to shellfish growers, and redistributing them to beaches where they are highly sought by tribal fishers would be a mutually beneficial solution for both parties. From a genomic standpoint, our analysis suggests that this would be a less risky solution than seeding beaches with captive‐bred individuals. Whereas captive‐bred cockles would have the potential to reduce the effective population size of the wild population, geoduck tube cockles exhibit comparable genomic profiles to those on natural beaches, including similar levels of *H*_o_, *H*_e_, *F*_IS_, *N_e_*, and low *F*_ST_.

The ultimate success of a *C. nuttallii* stock enhancement program is a key consideration. Fortunately, hatchery production has been extensively tested in *C. nuttallii*, and it has long been considered a prime candidate for aquaculture in the region due to ease of culture (Dunham et al., 2013a, 2013b; Liu, Alabi, & Pearce, 2008a, 2008b; Liu et al., 2009, 2010, 2011). Pilot breeding projects by our group have produced large quantities of *C. nuttallii* seed (>1 M) at relatively low cost and effort (Puget Sound Restoration Fund, unpublished). Typical seeding densities of Manila clams in Puget Sound are 300–600 m^−2^ (Toba, 1992), and similar densities have been used successfully in cockle beach culture pilot studies (600 m^−2^; Brooks, 2001). Meanwhile, the wild seed source available in geoduck tubes would be more cost‐effective and pose fewer genetic risks, yet it is unclear how much of this resource exists. However, geoduck farms cover approximately 80 ha in Puget Sound and the potential cockle biomass in these farms is significant; surveys to estimate this biomass are planned for the near future. We suspect that, owing to both their wild origin and larger size, geoduck tube cockles would be more successful than hatchery‐raised seed. Some studies of shellfish stock enhancement programs have found limited efficacy using hatchery‐bred seed, with potential explanations including predation and artificial selection (e.g., Carlsson et al., 2008). Cockles from geoduck tubes may be less prone to these effects due to their larger size and wild origin, and could be planted at densities approximating the highest observed natural densities in Puget Sound (232 g m^−3^, equivalent to ~6 cockles @ 50 mm; Barber et al., 2019).

## 5 CONCLUSIONS

Our analysis suggests that *C. nuttallii* populations in the southern Salish Sea are well connected and have strong potential to adapt and recover from disturbances. We cannot therefore recommend a large‐scale stock enhancement strategy that would risk undermining the natural genetic diversity of the species, and instead suggest either a small enhancement program or a sea farming approach that limits reproductive contributions of captive‐bred individuals. Meanwhile, questions remain regarding the causes of localized declines and slow recovery in certain areas. Several characteristics of *C. nuttallii*, such as shallow sediment burial depth, relatively short life span, and high fecundity, may make it particularly susceptible to boom‐and‐bust cycles, which could be amplified by climate change. This highlights the disproportionate vulnerability to climate change of Indigenous populations that rely heavily on nearshore subsistence harvests (Donatuto et al., 2014; Weatherdon et al., 2016). We advocate for continued monitoring of *C. nuttallii* populations in the region, including research on causes of declines in certain areas, and potential reevaluation of the need for stock enhancement in coming decades.

## Supporting information

Fig S1‐S6

## Data Availability

Demultiplexed sequence reads are available under NCBI BioProject PRJNA771709 (https://www.ncbi.nlm.nih.gov/bioproject/PRJNA771709/) (Dimond, 2021).
