# Supplementary material for: Population genomics of the basket cockle Clinocardium nuttallii in the southern Salish Sea: Assessing genetic risks of stock enhancement for a culturally important marine bivalve
Source: Evol Appl. 2022 Mar 8;15(3):459–70. doi: 10.1111/eva.13359 (PMC8965374; doi:10.1111/eva.13359)
Supplement: Supplementary file 1 — Fig S1‐S6 [file EVA-15-459-s001.docx]

Supplementary Figures


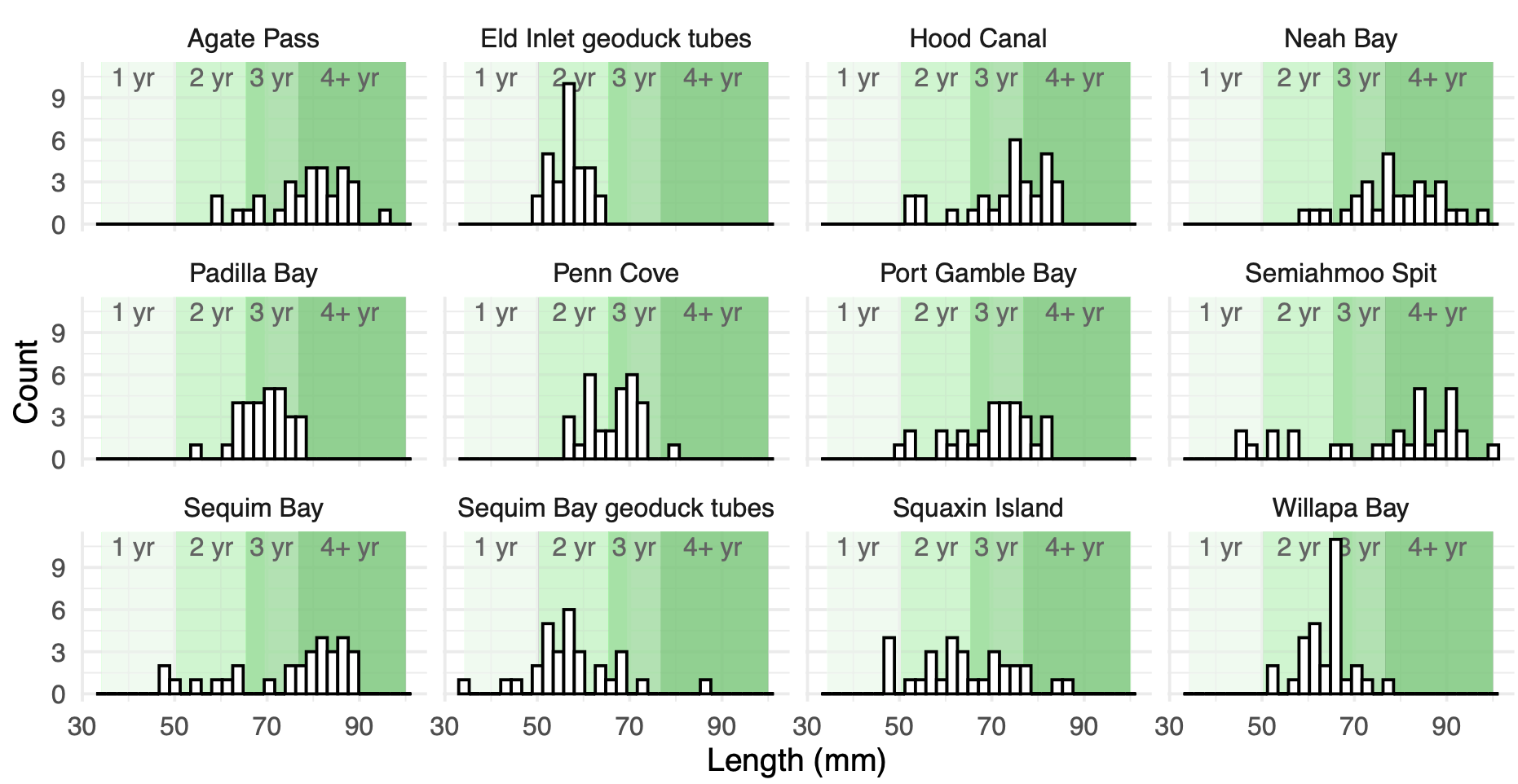


Figure S1. Shell lengths of *C. nuttallii* collected at each site. Shading indicates approximate ages of cockles based on length-age data reported by [(Gallucci & Gallucci, 1982)](https://sciwheel.com/work/citation?ids=7620978&pre=&suf=&sa=0).


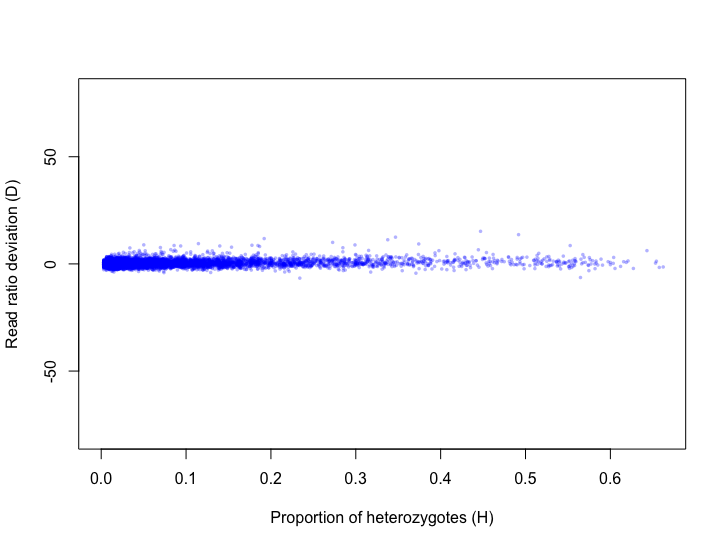


Figure S2. Identification of putative paralogous loci using the proportion of heterozygotes vs. read ratio deviation procedure [(McKinney et al., 2017)](https://sciwheel.com/work/citation?ids=2712268&pre=&suf=&sa=0)). The clustering of all loci at or near zero on the y-axis suggests that no paralogs were present in the final assembly.


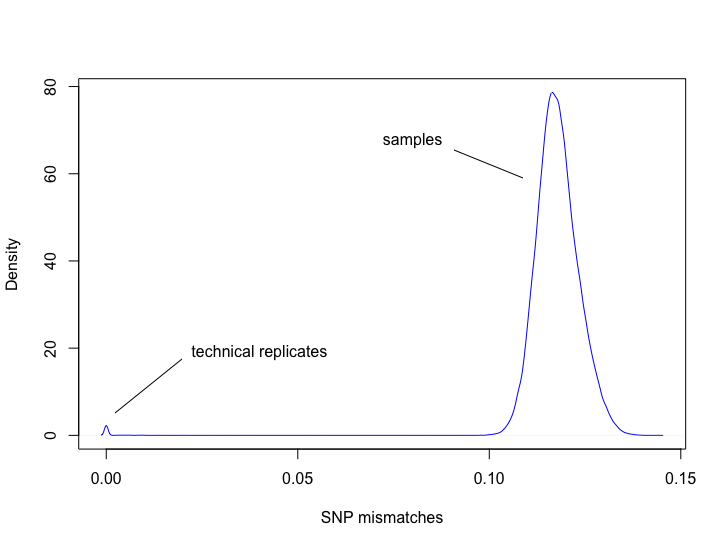


Figure S3. Density plot of all pairwise SNP mismatch comparisons (genetic distance). Samples sequenced in duplicate were used as technical replicates to estimate genotyping error, which was 0.72%. By contrast, all SNP mismatches between samples exceeded 10%.


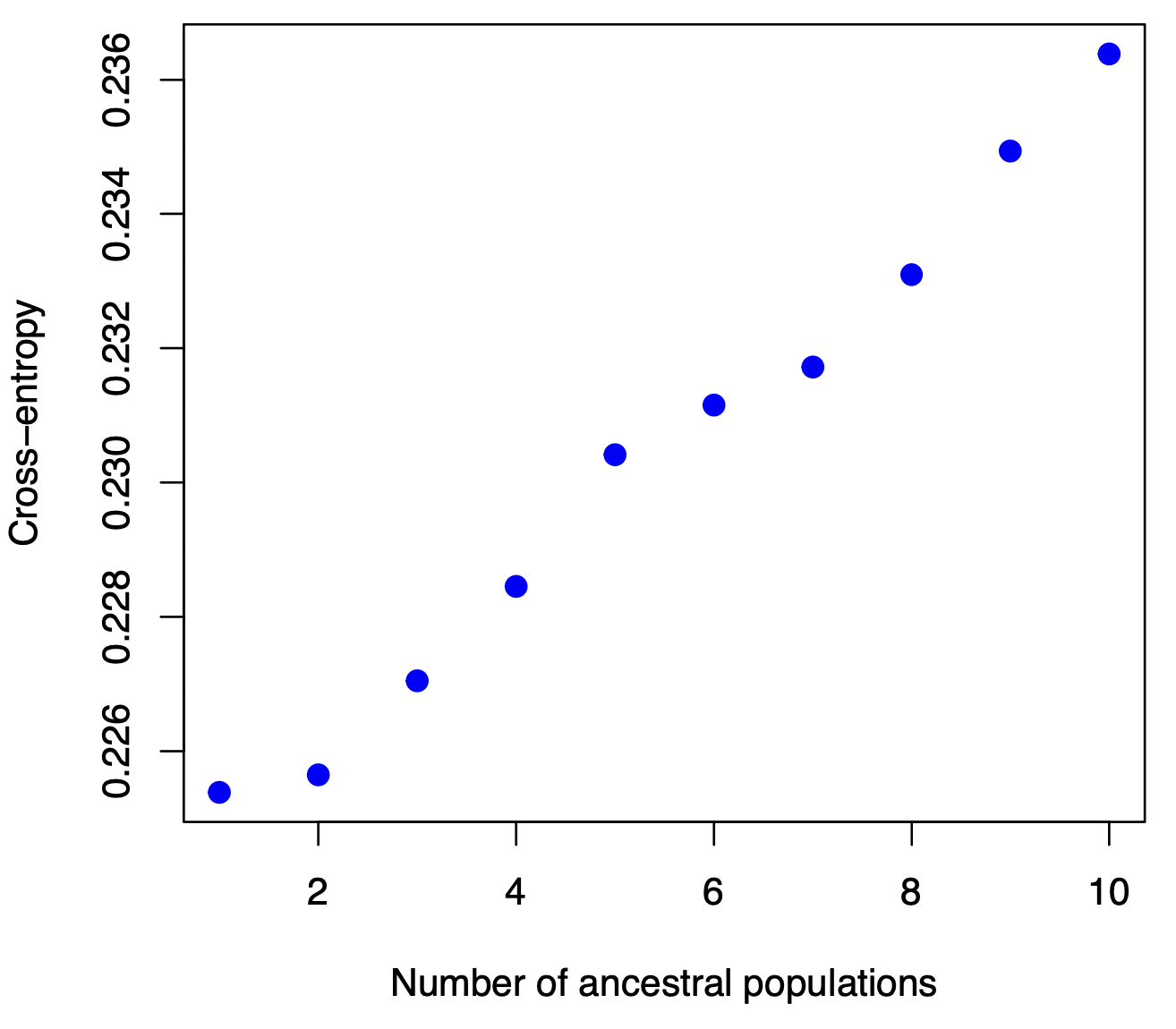


Figure S4. Cross-entropy values for *K =* 1-10 ancestral populations.


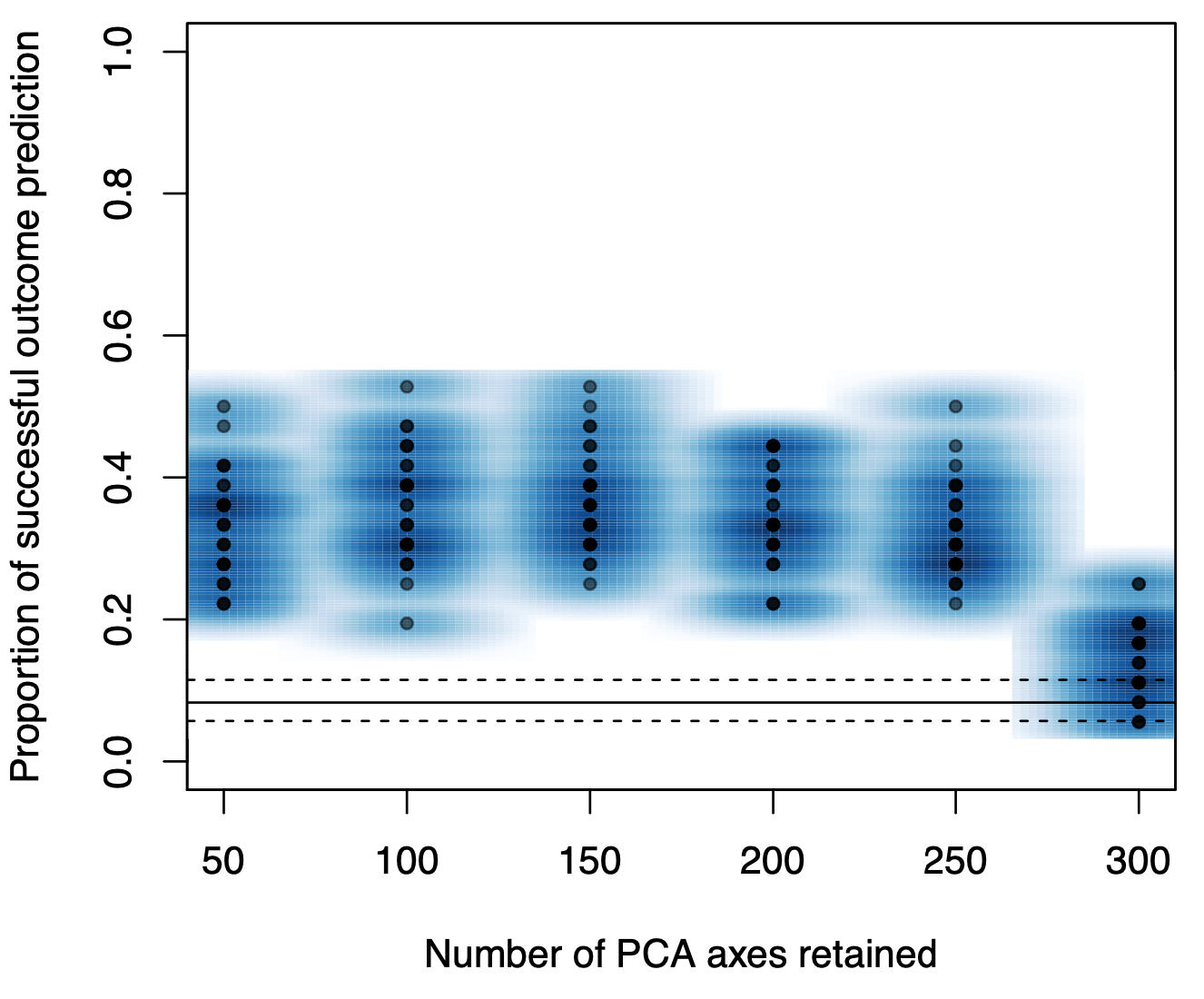


Figure S5. Results of discriminant analysis of principal components (DAPC) cross-validation identifying the optimal number of principal components for the DAPC.

*
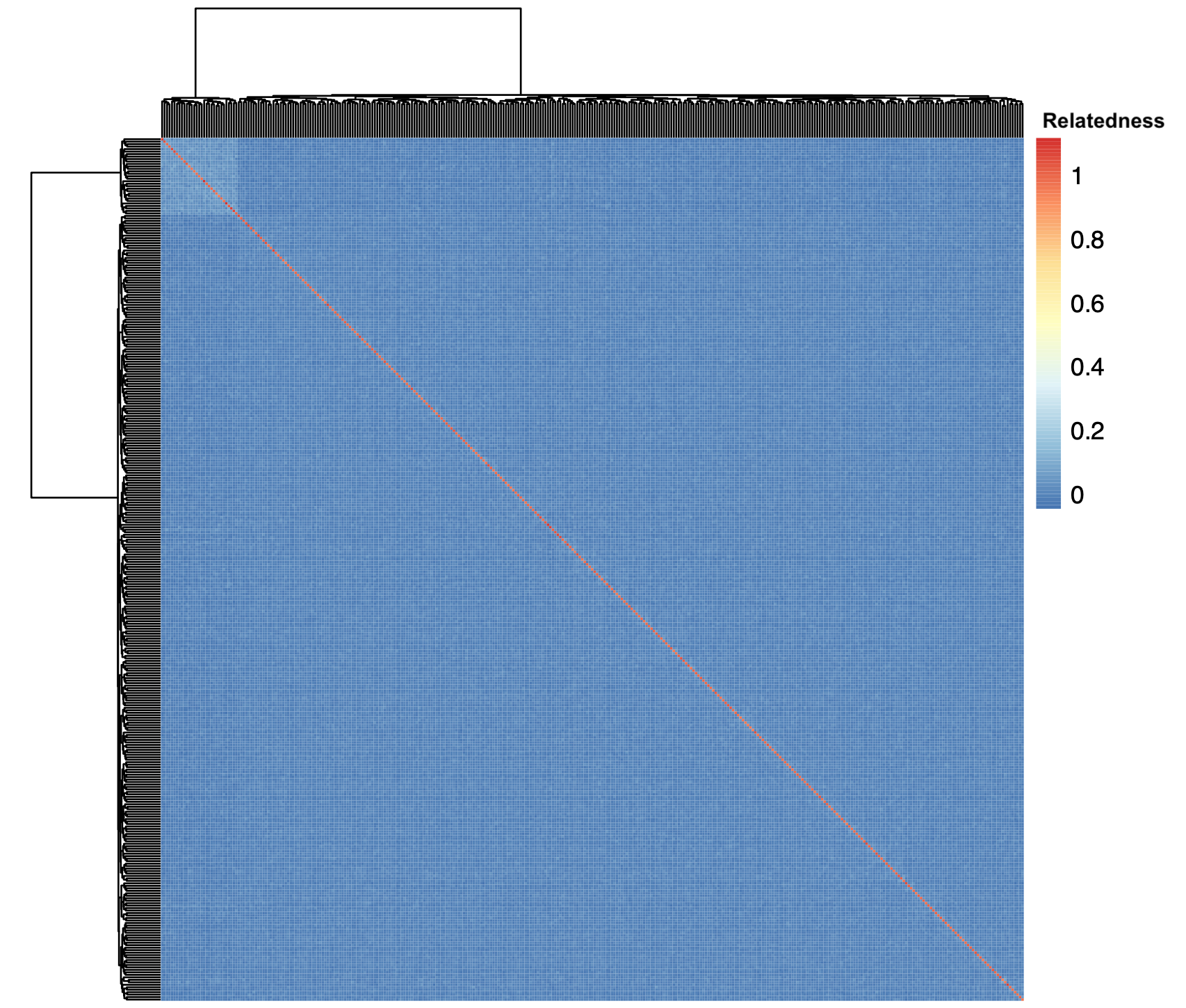
*

Figure S6. Genomic relatedness matrix for all individuals (n = 349). Individuals are shown in both rows and columns, with ordering via hierarchical clustering. Willapa Bay individuals appear in the slightly lighter
